# Supplementary material for: Knowledge-enhanced visual-language pre-training on chest radiology images
Source: Nat Commun. 2023 Jul 28;14:4542. doi: 10.1038/s41467-023-40260-7 (PMC10382552; doi:10.1038/s41467-023-40260-7)
Supplement: Supplementary file 1 — Supplementary Information [file 41467_2023_40260_MOESM1_ESM.pdf]

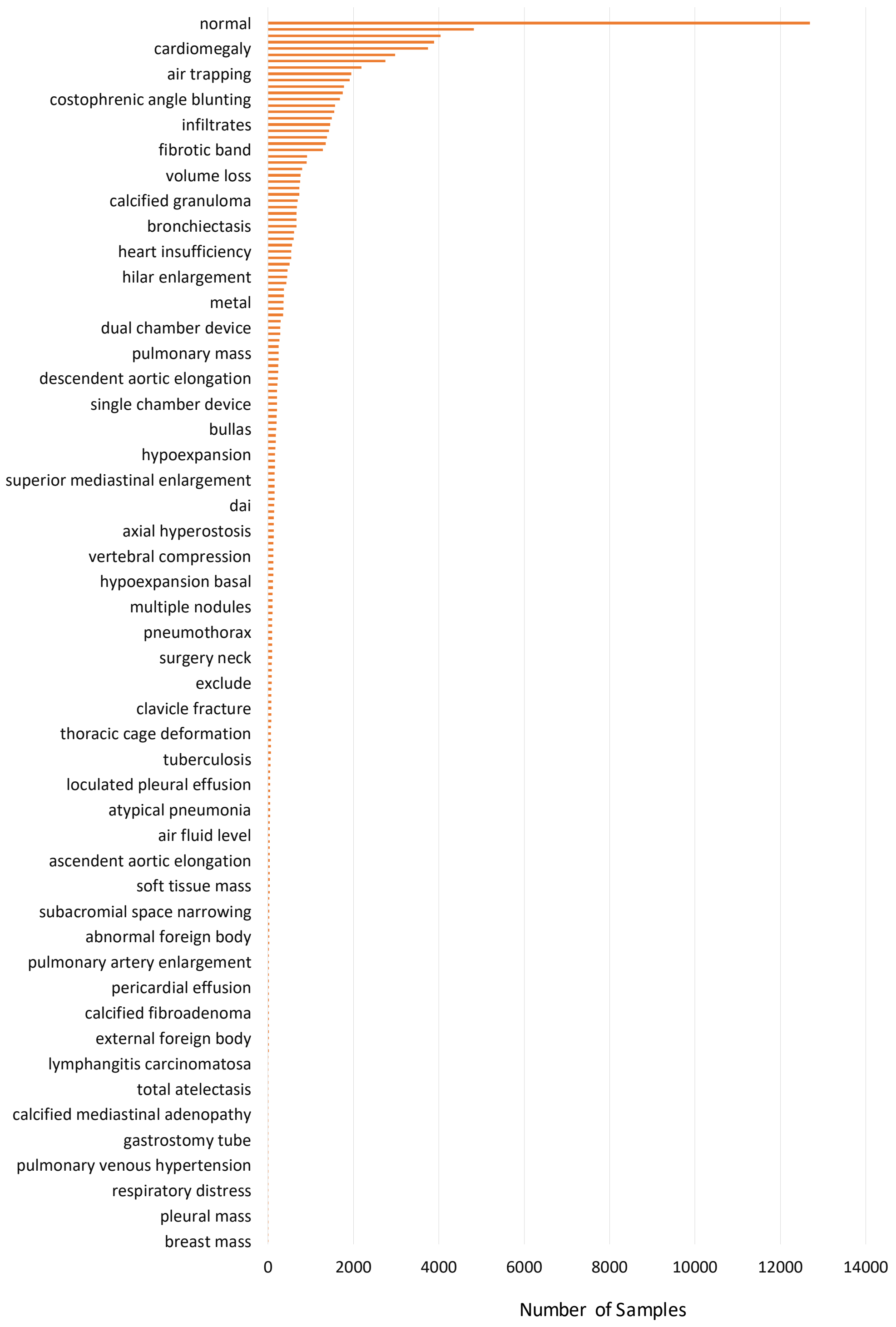

Supplementary Fig. 1: Dataset distribution of PadChest (39053 manually labeled samples). It can be observed that only 21 classes have more than 1000 samples and the data tend to show a long-tailed distribution.

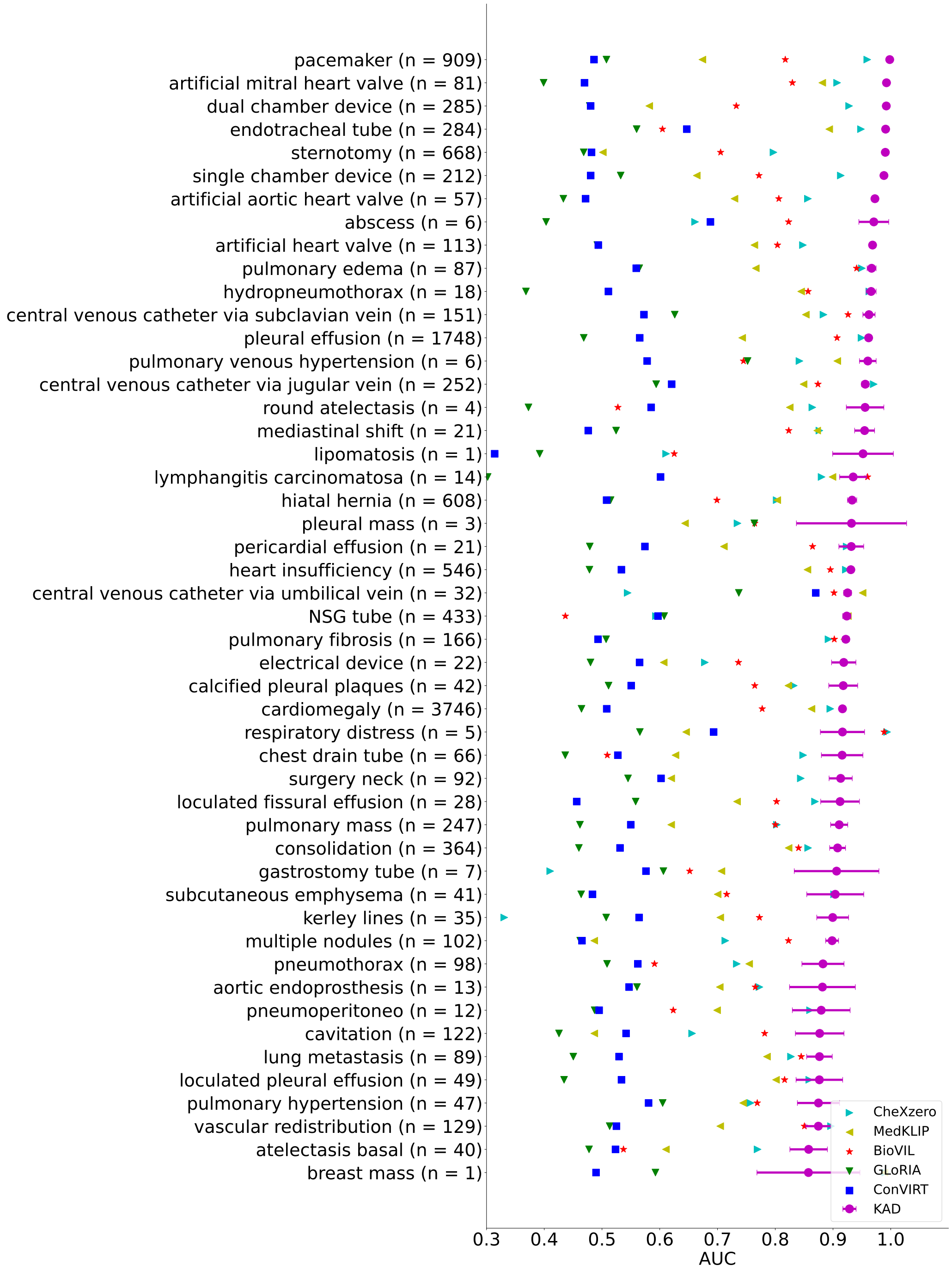

Supplementary Fig. 2: Comparison of KAD with SOTA medical image-text pre-training models on the 174 different radiographic findings and 19 differential diagnoisis, totaling 193 classes. We evaluate model on the human-annotated subset of the PadChest dataset (n = 39,053 chest X-rays) under **zero-shot** setting. Here we show the results of the results for the **1-49** classes. Mean AUC and 95% CI of KAD are shown for each class, and n refers to the number of positive samples.

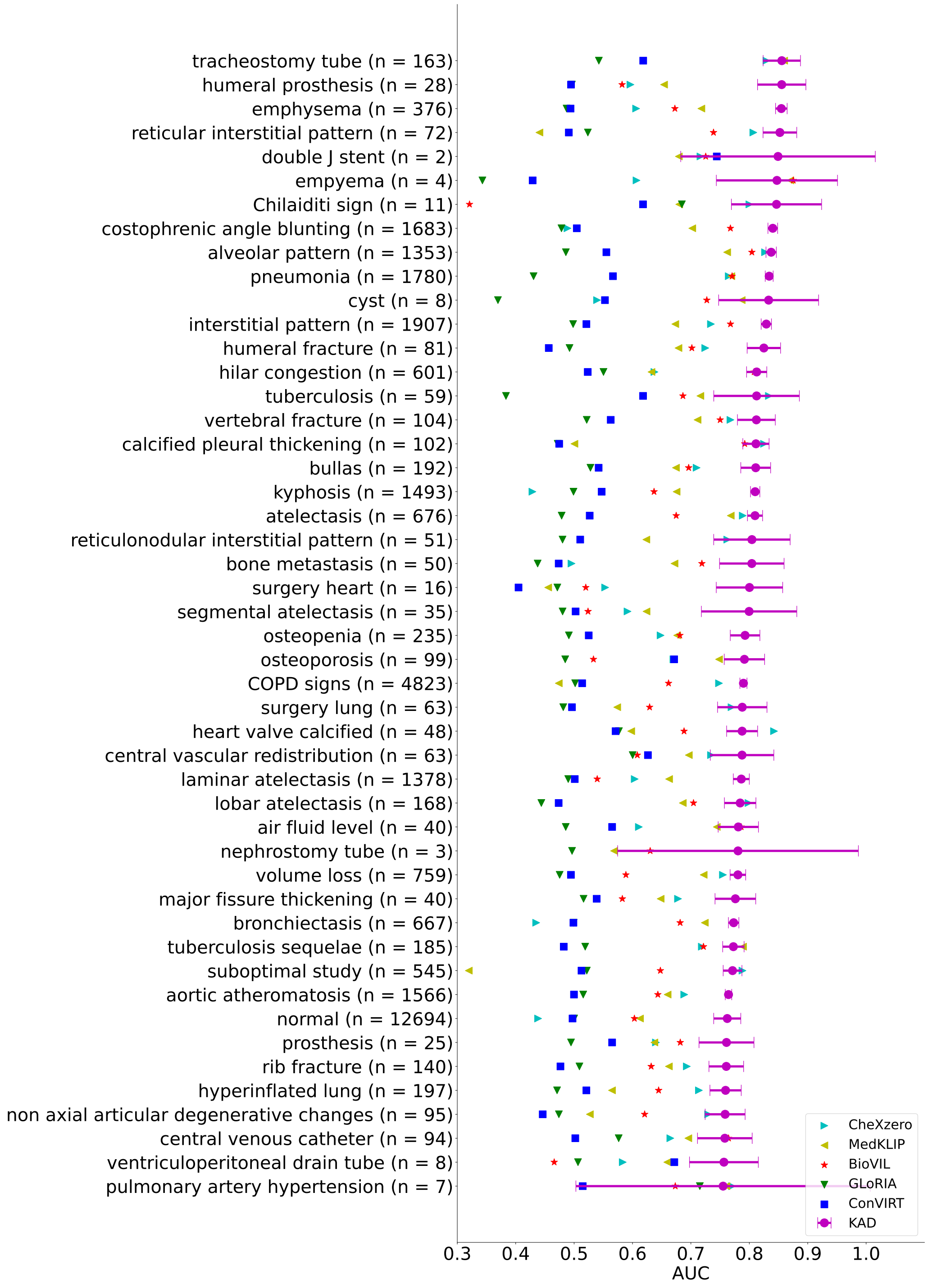

Supplementary Fig. 3: Comparison of KAD with SOTA medical image-text pre-training models on the 174 different radiographic findings and 19 differential diagnoisis, totaling 193 classes. We evaluate model on the human-annotated subset of the PadChest dataset (n = 39,053 chest X-rays) under **zero-shot** setting. Here we show the results of the results for the **50-97** classes. Mean AUC and 95% CI of KAD are shown for each class, and n refers to the number of positive samples.

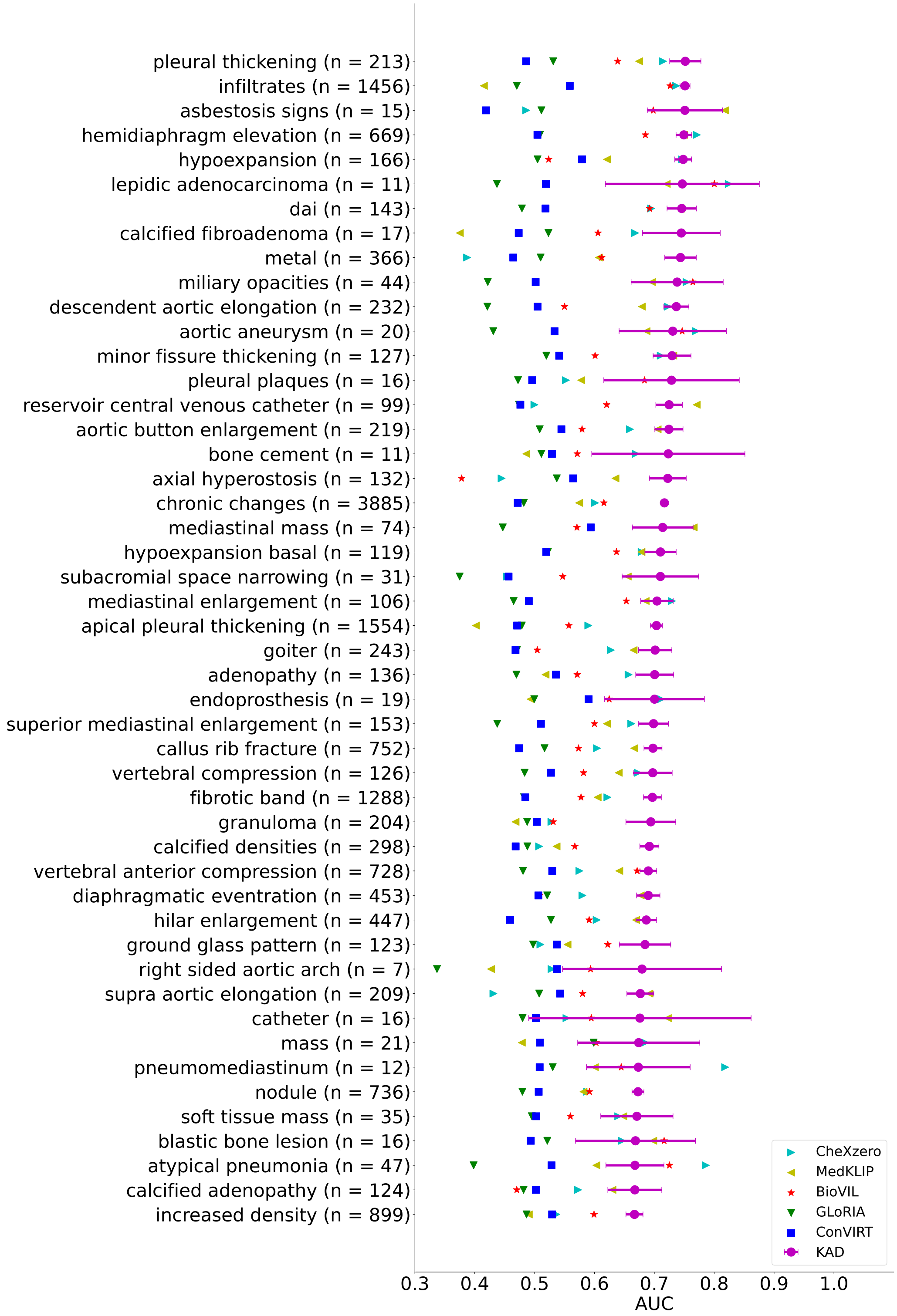

Supplementary Fig. 4: Comparison of KAD with SOTA medical image-text pre-training models on the 174 different radiographic findings and 19 differential diagnoisis, totaling 193 classes. We evaluate model on the human-annotated subset of the PadChest dataset (n = 39,053 chest X-rays) under **zero-shot** setting. Here we show the results of the results for the **98-145** classes. Mean AUC and 95% CI of KAD are shown for each class, and n refers to the number of positive samples.

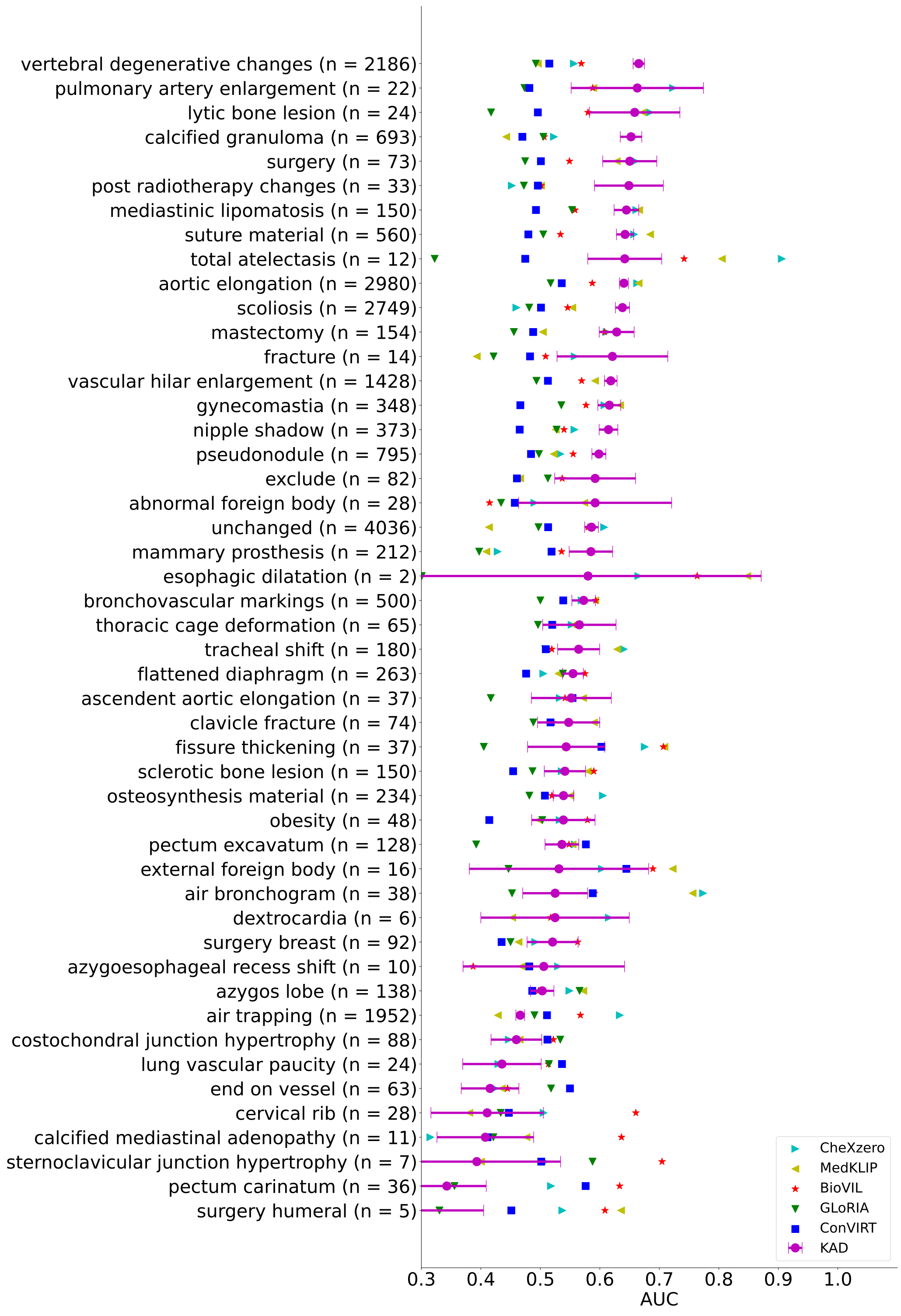

Supplementary Fig. 5: Comparison of KAD with SOTA medical image-text pre-training models on the 174 different radiographic findings and 19 differential diagnoisis, totaling 193 classes. We evaluate model on the human-annotated subset of the PadChest dataset (n = 39,053 chest X-rays) under **zero-shot** setting. Here we show the results of the results for the **146-193** classes. Mean AUC and 95% CI of KAD are shown for each class, and n refers to the number of positive samples.

Supplementary Table 1: Comparison with other state-of-the-art medical image-text pre-training methods on **zero-shot** classification task on ChestX-ray14.

Metric	Method	Mean	Atelectasis	Cardiomegaly	Effusion	Infiltration	Mass	Nodule	Pneumonia	Pneumothorax	Consolidation	Edema	Emphysema	Fibrosis	Pleural Thicken	Hernia
AUC	ConVIRT	0.560	0.459	0.433	0.646	0.654	0.601	0.580	0.640	0.533	0.646	0.692	0.431	0.482	0.545	0.494
	GLoRIA	0.610	0.653	0.704	0.762	0.660	0.613	0.508	0.587	0.572	0.697	0.762	0.499	0.459	0.613	0.450
	BioViL	0.662	0.517	0.688	0.743	0.601	0.663	0.639	0.669	0.683	0.650	0.795	0.656	0.632	0.637	0.698
	MedKLIP	0.726	0.671	0.842	0.813	**0.706**	0.742	0.621	0.698	0.821	**0.719**	0.803	0.783	0.604	0.499	0.841
	KAD	**0.789**	**0.770**	**0.854**	**0.824**	0.694	**0.754**	**0.698**	**0.734**	**0.860**	0.718	**0.809**	**0.879**	**0.780**	0.718	**0.952**
	(95% CI)	0.770	0.750	0.839	0.816	0.679	0.736	0.676	0.712	0.850	0.703	0.780	0.866	0.752	0.698	0.917
		0.808	0.790	0.869	0.832	0.709	0.772	0.719	0.756	0.870	0.733	0.838	0.892	0.808	0.740	0.986
MCC	ConVIRT	0.074	0.004	0.013	0.182	0.207	0.100	0.071	0.066	0.079	0.113	0.119	0.012	0.009	0.049	0.014
	GLoRIA	0.108	0.157	0.126	0.313	0.210	0.087	0.021	0.044	0.095	0.156	0.167	0.032	0.008	0.076	0.016
	BioViL	0.133	0.056	0.173	0.289	0.138	0.124	0.102	0.076	0.180	0.138	0.207	0.102	0.058	0.086	0.124
	MedKLIP	0.197	0.171	0.273	0.396	**0.282**	0.203	0.106	0.092	0.371	**0.190**	0.199	0.238	0.066	0.018	0.154
	KAD	**0.280**	**0.293**	**0.344**	**0.419**	0.270	**0.237**	**0.155**	**0.114**	**0.445**	0.174	**0.200**	**0.389**	**0.149**	**0.142**	**0.582**
	(95% CI)	0.248	0.258	0.302	0.400	0.246	0.206	0.125	0.098	0.420	0.159	0.180	0.373	0.122	0.125	0.463
		0.312	0.328	0.386	0.438	0.294	0.268	0.185	0.129	0.469	0.188	0.219	0.424	0.175	0.158	0.700
F1	ConVIRT	0.135	0.001	0.002	0.367	0.436	0.157	0.142	0.060	0.205	0.177	0.121	0.083	0.034	0.097	0.007
	GLoRIA	0.174	0.281	0.167	0.452	0.442	0.155	0.122	0.053	0.209	0.200	0.146	0.086	0.004	0.109	0.007
	BioViL	0.192	0.235	0.209	0.438	0.414	0.178	0.164	0.067	0.274	0.177	0.187	0.123	0.056	0.119	0.045
	MedKLIP	0.244	0.292	0.301	0.516	**0.483**	0.256	0.175	0.076	0.438	**0.218**	**0.173**	0.246	0.079	0.010	0.154
	KAD	**0.323**	**0.392**	**0.359**	**0.534**	0.475	**0.296**	**0.210**	**0.086**	**0.499**	0.196	0.170	**0.424**	**0.128**	**0.163**	**0.580**
	(95% CI)	0.294	0.363	0.318	0.518	0.459	0.266	0.181	0.074	0.478	0.185	0.151	0.399	0.106	0.150	0.462
		0.351	0.420	0.400	0.550	0.490	0.326	0.238	0.098	0.520	0.207	0.188	0.448	0.150	0.175	0.698
ACC	ConVIRT	0.459	0.872	**0.958**	0.504	0.632	0.389	0.494	0.520	0.266	0.548	0.661	0.063	0.029	0.354	0.132
	GLoRIA	0.503	0.466	0.919	0.674	0.613	0.447	0.183	0.482	0.279	0.586	0.694	0.183	**0.982**	0.466	0.072
	BioViL	0.633	0.218	0.904	0.678	0.403	0.571	0.644	0.604	0.648	0.437	**0.790**	0.615	0.747	0.601	0.997
	MedKLIP	0.796	0.520	0.919	0.799	**0.672**	0.809	0.825	0.671	0.837	**0.607**	0.754	0.858	0.924	**0.954**	0.993
	KAD	**0.816**	**0.799**	0.956	**0.802**	0.671	**0.878**	**0.896**	**0.742**	**0.862**	0.461	0.733	**0.952**	0.906	0.768	**0.997**
	(95% CI)	0.810	0.790	0.952	0.796	0.660	0.871	0.890	0.738	0.855	0.455	0.724	0.949	0.899	0.759	0.996
		0.822	0.807	0.959	0.807	0.681	0.885	0.901	0.747	0.869	0.467	0.741	0.955	0.912	0.777	0.998

AUC, MCC, F1 and ACC scores are reported, and the metrics all refer to the macro average on all the diseases. Numbers within the last two rows indicate 95% CI. The best results are bold.

Supplementary Table 2: Comparison with other state-of-the-art medical image-text pre-training and self-supervised learning methods on fine-tuning classification task on ChestX-ray14.

Training data	Method	Mean	Atelectasis	Cardiomegaly	Effusion	Infiltration	Mass	Nodule	Pneumonia	Pneumothorax	Consolidation	Edema	Emphysema	Fibrosis	Pleural Thicken	Hernia
1%	ResNet-50	0.581	0.557	0.577	0.636	0.616	0.550	0.602	0.571	0.582	0.608	0.633	0.534	0.637	0.568	0.460
	ConVIRT	0.649	0.660	0.782	0.789	0.611	0.596	0.655	0.608	0.688	0.657	0.607	0.658	0.680	0.627	0.466
	GLoRIA	0.597	0.597	0.567	0.741	0.646	0.559	0.557	0.611	0.607	0.665	0.669	0.550	0.558	0.592	0.436
	BioViL	0.579	0.555	0.564	0.722	0.650	0.567	0.546	0.626	0.560	0.657	0.681	0.516	0.513	0.592	0.360
	MG	0.595	0.604	0.741	0.705	0.608	0.514	0.597	0.557	0.544	0.645	0.670	0.505	0.669	0.581	0.395
	C2L	0.613	0.650	0.644	0.753	0.640	0.572	0.585	0.632	0.540	0.654	0.706	0.496	0.634	0.593	0.485
	ImageNet	0.635	0.662	0.642	0.721	0.570	0.590	0.585	0.600	0.626	0.624	0.668	0.615	0.707	0.631	0.645
	MedKLIP	0.609	0.655	0.590	0.745	0.643	0.550	0.611	0.609	0.599	0.659	0.682	0.535	0.648	0.593	0.400
	KAD	**0.787**	**0.770**	**0.882**	**0.829**	**0.692**	**0.751**	**0.697**	**0.735**	**0.861**	**0.727**	**0.813**	**0.893**	**0.743**	**0.692**	**0.938**
10%	ResNet-50	0.691	0.682	0.766	0.746	0.674	0.623	0.580	0.636	0.728	0.678	0.780	0.647	0.715	0.653	0.771
	ConVIRT	0.771	0.740	0.843	0.811	0.693	0.748	0.700	0.671	0.828	0.701	0.814	0.871	0.767	0.719	0.893
	GLoRIA	0.743	0.721	0.808	0.800	0.687	0.733	0.675	0.658	0.779	0.676	0.797	0.799	0.787	0.693	0.787
	BioViL	0.727	0.703	0.785	0.790	0.666	0.718	0.671	0.665	0.767	0.684	0.799	0.761	0.748	0.653	0.763
	MG	0.703	0.697	0.798	0.774	0.663	0.666	0.594	0.604	0.708	0.682	0.770	0.645	0.735	0.665	0.841
	C2L	0.732	0.714	0.834	0.789	0.684	0.687	0.654	0.631	0.786	0.701	0.794	0.774	0.742	0.679	0.776
	ImageNet	0.726	0.709	0.798	0.769	0.684	0.693	0.656	0.630	0.793	0.671	0.767	0.749	0.729	0.711	0.810
	MedKLIP	0.748	0.729	0.802	0.793	0.698	0.719	0.681	0.666	0.796	0.696	0.811	0.795	0.756	0.713	0.819
	KAD	**0.807**	**0.776**	**0.889**	**0.833**	**0.718**	**0.783**	**0.719**	**0.737**	**0.872**	**0.750**	**0.833**	**0.903**	**0.807**	**0.723**	**0.953**
100%	ResNet-50	0.790	0.750	0.879	0.815	0.691	0.798	0.726	0.703	0.826	0.731	0.839	0.835	0.807	0.754	0.903
	ConVIRT	0.808	0.771	0.867	0.825	0.703	0.818	0.761	0.722	0.857	0.747	0.854	0.901	0.809	0.771	0.909
	GLoRIA	0.800	0.760	0.855	0.818	0.700	0.814	0.749	0.715	0.828	0.739	0.832	0.887	0.813	0.767	0.921
	BioViL	0.800	0.765	0.871	0.824	0.697	0.819	0.752	0.710	0.845	0.742	0.842	0.871	0.821	0.759	0.888
	MG	0.777	0.737	0.866	0.812	0.677	0.786	0.674	0.689	0.806	0.733	0.834	0.833	0.797	0.744	0.892
	C2L	0.802	0.766	0.865	0.824	0.688	0.810	0.758	0.712	0.845	0.744	0.830	0.889	0.811	0.763	0.925
	ImageNet	0.804	0.763	0.867	0.823	0.693	0.823	0.763	0.719	0.840	0.737	0.842	0.893	0.819	0.770	0.899
	MedKLIP	0.801	0.764	0.849	0.823	0.697	0.820	0.747	0.712	0.839	0.751	0.848	0.879	0.817	0.777	0.892
	KAD	**0.825**	**0.785**	**0.897**	**0.840**	**0.713**	**0.836**	**0.771**	**0.740**	**0.874**	**0.753**	**0.860**	**0.916**	**0.829**	**0.778**	**0.961**

AUC scores are reported, and the metrics all refer to the macro average on all the diseases. The best results are bold. MG refers to "Model Genesis". ImageNet refers to "ImageNet Pre-training".

Supplementary Table 3: Comparisons of proposed KAD with SOTA medical image-text pre-training models and three board-certified radiologists on five competition pathologies in CheXpert dataset.

	Mean	Atelectasis	Cardiomegaly	Consolidation	Edema	Pleural effusion
**AUC**						
ConVIRT	0.590 (0.557,0.624)	0.524 (0.512,0.536)	0.548 (0.525,0.571)	0.680 (0.630,0.730)	0.582 (0.537,0.626)	0.618 (0.580,0.656)
GLoRIA	0.750 (0.711,0.789)	0.807 (0.779,0.836)	0.802 (0.781,0.823)	0.588 (0.540,0.635)	0.747 (0.687,0.806)	0.807 (0.769,0.846)
BioVIL	0.693 (0.651,0.736)	0.637 (0.604,0.671)	0.694 (0.671,0.716)	0.705 (0.636,0.774)	0.638 (0.601,0.675)	0.793 (0.743,0.844)
CheXzero	0.889 (0.849,0.922)	0.816 (0.777,0.852)	**0.906** (0.876,0.930)	**0.892** (0.823,0.947)	0.897 (0.864,0.928)	0.932 (0.906,0.955)
MedKLIP	0.879 (0.855,0.903)	0.813 (0.790,0.835)	0.866 (0.842,0.890)	0.858 (0.821,0.895)	0.911 (0.890,0.931)	0.947 (0.931,0.963)
KAD	**0.905** (0.886,0.924)	**0.884** (0.863,0.904)	0.885 (0.866,0.904)	0.865 (0.837,0.893)	**0.943** (0.928,0.958)	**0.949** (0.935,0.963)
**MCC**						
Radiologists(mean)	0.530 (0.499,0.558)	0.548 (0.496,0.606)	0.566 (0.511,0.620)	0.359 (0.262,0.444)	0.507 (0.431,0.570)	0.548 (0.496,0.606)
ConVIRT	0.231 (0.150,0.313)	0.180 (0.132,0.229)	0.146 (0.081,0.211)	0.315 (0.225,0.405)	0.280 (0.154,0.405)	0.236 (0.159,0.314)
GLoRIA	0.501 (0.419,0.584)	0.550 (0.502,0.598)	0.610 (0.568,0.651)	0.275 (0.142,0.408)	0.443 (0.313,0.574)	0.628 (0.567,0.688)
BioVIL	0.368 (0.297,0.438)	0.249 (0.191,0.306)	0.439 (0.379,0.499)	0.252 (0.165,0.340)	0.403 (0.332,0.474)	0.495 (0.419,0.571)
CheXzero	0.523 (0.486,0.561)	0.468 (0.396,0.541)	**0.625** (0.553,0.700)	0.374 (0.290,0.458)	0.520 (0.424,0.616)	0.628 (0.558,0.696)
MedKLIP	0.540 (0.476,0.603)	0.476 (0.434,0.517)	0.574 (0.522,0.625)	**0.404** (0.295,0.512)	0.563 (0.502,0.623)	0.683 (0.627,0.739)
KAD	**0.589** (0.536,0.642)	**0.613** (0.567,0.659)	0.607 (0.558,0.656)	0.357 (0.293,0.421)	**0.666** (0.608,0.724)	**0.702** (0.653,0.751)
**F1**						
Radiologists(mean)	0.619 (0.585,0.642)	0.692 (0.646,0.731)	0.678 (0.634,0.718)	0.385 (0.280,0.485)	0.583 (0.511,0.645)	0.737 (0.689,0.783)
ConVIRT	0.264 (0.187,0.342)	0.090 (0.045,0.135)	0.234 (0.176,0.292)	0.353 (0.261,0.444)	0.272 (0.151,0.393)	0.373 (0.301,0.445)
GLoRIA	0.570 (0.498,0.643)	0.668 (0.636,0.699)	0.713 (0.675,0.751)	0.269 (0.145,0.393)	0.511 (0.391,0.630)	0.691 (0.641,0.742)
BioVIL	0.463 (0.408,0.518)	0.496 (0.461,0.531)	0.545 (0.505,0.585)	0.276 (0.204,0.347)	0.407 (0.327,0.487)	0.590 (0.542,0.639)
CheXzero	0.606 (0.571,0.638)	0.646 (0.593,0.700)	**0.743** (0.685,0.793)	0.333 (0.239,0.424)	0.602 (0.517,0.678)	0.704 (0.634,0.764)
MedKLIP	0.614 (0.555,0.673)	0.624 (0.589,0.658)	0.694 (0.656,0.732)	**0.415** (0.300,0.529)	0.601 (0.541,0.661)	0.738 (0.689,0.787)
KAD	**0.646** (0.591,0.702)	**0.720** (0.681,0.758)	0.721 (0.684,0.757)	0.342 (0.237,0.447)	**0.701** (0.647,0.754)	**0.748** (0.705,0.792)

Note that, all models are directly evaluated on CheXpert dataset under **zero-shot** setting. Numbers within parentheses indicate 95% CI. The best results are bold.

Supplementary Table 4: Comparisons of proposed KAD with SOTA medical image-text pre-training models on zero-shot classification and grounding task in ChestX-Det10 dataset.

Metric	Method	Mean	Atelectasis	Calcification	Consolidation	Effusion	Emphysema	Fibrosis	Fracture	Mass	Nodule	Pneumothorax
AUC	GLoRIA	0.645	0.622	0.524	0.718	0.866	0.607	0.523	0.494	0.725	0.63	0.744
	BioViL	0.663	0.617	0.521	0.808	0.789	0.78	0.607	0.623	0.612	0.591	0.686
	KAD	0.735	0.757	0.563	0.824	0.888	0.888	0.687	0.608	0.695	0.566	0.874
	KAD-512	0.771	0.812	0.578	0.854	0.892	0.916	0.671	0.666	0.703	0.705	0.913
	KAD-1024	0.764	0.793	0.661	0.789	0.861	0.880	0.684	0.606	0.680	0.763	0.923
Pointing Game	GLoRIA	0.367	0.479	0.053	**0.737**	0.528	0.667	0.366	0.013	**0.533**	0.156	0.143
	BioViL	0.380	0.375	0.105	0.664	0.615	**0.795**	0.378	0.013	0.500	0.130	0.229
	KAD	0.391	0.646	0.132	0.699	0.618	0.644	0.244	0.199	0.267	0.316	0.143
	KAD-512	0.462	0.729	0.158	0.713	**0.738**	0.769	0.293	**0.237**	0.433	0.377	0.171
	KAD-1024	**0.485**	**0.771**	**0.316**	0.692	0.560	0.718	**0.379**	0.132	0.367	**0.571**	**0.343**

AUC scores are shown for the zero-shot classification task, and Pointing game scores are shown for the zero-shot grounding task. Note that, all models are directly evaluated on ChestX-Det10 dataset under **zero-shot** setting. The best results are bold.

```python
import spacy
from collections import defaultdict
# "en_core_sci_lg" is a full spacy pipeline for biomedical data with a larger vocabulary
                                              and 600k word vectors
nlp = spacy.load("en_core_sci_lg")
nlp.add_pipe("abbreviation_detector")
# "scispacy_linker" is a spacy component that performs linking to a knowledge base~(UMLS
                                              in our case)
nlp.add_pipe("scispacy_linker",
            config={"resolve_abbreviations": True, "linker_name": "umls"})
sentence_entities = [] #sentence_entities refers the extracted list
caption_nlp = nlp(caption) # caption refers to the sentence of the radiology report
entities = caption_nlp.ents
for entity in entities:
    entity_dict = defaultdict(list)
    linker = nlp.get_pipe("scispacy_linker")
    for umls_ent in entity._.kb_ents:
        umls_ent_info = linker.kb.cui_to_entity[umls_ent[0]]
        entity_dict['entity'] = entity
        entity_dict['concept'] = umls_ent_info[1]
        entity_dict['CUI'] = umls_ent_info[0]
        entity_dict['TUI'] = umls_ent_info[3]
    sentence_entities.append(entity_dict)
```

Supplementary Fig. 6: Pseudo code for entity extraction using the Unified Medical Language System.

Supplementary Table 5: Entity set $\mathcal{Q}$. We select top $Q = 40$ most commonly appearing entities in the report corpus.

pleural effusion	opacity	pneumothorax	edema
atelectasis	tube	consolidation	enlarged cardiomediastinum
tip	pneumonia	line	cardiomegaly
fracture	calcification	device	engorgement
nodule	wire	pacemaker	pleural thicken
marking	scar	hyperinflate	blunt
collapse	emphysema	aerate	mass
infiltration	obscure	deformity	hernia
drainage	distention	shift	normal
lesion	hardware	dilation	aspiration

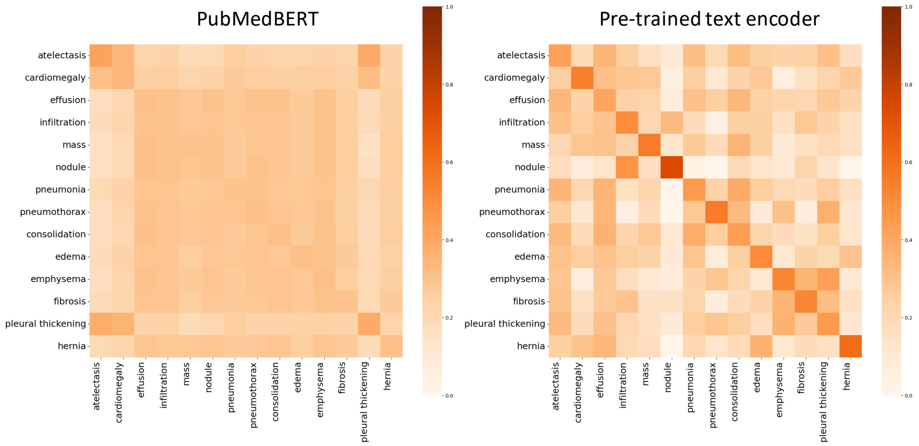

Supplementary Fig. 7: Similarity map between features of different disease names encoded by PubMed-BERT and Med-KEBERT.
