# Supplementary material for: Knowledge-enhanced visual-language pre-training on chest radiology images
Source: Nat Commun. 2023 Jul 28;14:4542. doi: 10.1038/s41467-023-40260-7 (PMC10382552; doi:10.1038/s41467-023-40260-7)
Supplement: Supplementary file 3 — Reporting Summary [file 41467_2023_40260_MOESM3_ESM.pdf]

nature portfolio

Corresponding author(s): Weidi Xie, Yanfeng Wang

Last updated by author(s): Jul 1, 2023

Reporting Summary

Nature Portfolio wishes to improve the reproducibility of the work that we publish. This form provides structure for consistency and transparency in reporting. For further information on Nature Portfolio policies, see our Editorial Policies and the Editorial Policy Checklist.

Statistics

For all statistical analyses, confirm that the following items are present in the figure legend, table legend, main text, or Methods section.

n/a	Confirmed	
[ ]	[x]	The exact sample size (*n*) for each experimental group/condition, given as a discrete number and unit of measurement
[ ]	[x]	A statement on whether measurements were taken from distinct samples or whether the same sample was measured repeatedly
[x]	[ ]	The statistical test(s) used AND whether they are one- or two-sided<br>*Only common tests should be described solely by name; describe more complex techniques in the Methods section.*
[x]	[ ]	A description of all covariates tested
[x]	[ ]	A description of any assumptions or corrections, such as tests of normality and adjustment for multiple comparisons
[ ]	[x]	A full description of the statistical parameters including central tendency (e.g. means) or other basic estimates (e.g. regression coefficient) AND variation (e.g. standard deviation) or associated estimates of uncertainty (e.g. confidence intervals)
[x]	[ ]	For null hypothesis testing, the test statistic (e.g. *F*, *t*, *r*) with confidence intervals, effect sizes, degrees of freedom and *P* value noted<br>*Give P values as exact values whenever suitable.*
[x]	[ ]	For Bayesian analysis, information on the choice of priors and Markov chain Monte Carlo settings
[x]	[ ]	For hierarchical and complex designs, identification of the appropriate level for tests and full reporting of outcomes
[x]	[ ]	Estimates of effect sizes (e.g. Cohen's *d*, Pearson's *r*), indicating how they were calculated

Our web collection on statistics for biologists contains articles on many of the points above.

Software and code

Policy information about availability of computer code

Data collection	No software was used for data collection.
Data analysis	The whole framework was implemented by python 3.9.12 and torch '1.9.1+cu111'. The software used in entity extraction module is scispacy v0.5.1. The code is available at https://github.com/xiaoman-zhang/KAD.

For manuscripts utilizing custom algorithms or software that are central to the research but not yet described in published literature, software must be made available to editors and reviewers. We strongly encourage code deposition in a community repository (e.g. GitHub). See the Nature Portfolio guidelines for submitting code & software for further information.

Data

Policy information about availability of data

All manuscripts must include a data availability statement. This statement should provide the following information, where applicable:

- Accession codes, unique identifiers, or web links for publicly available datasets
- A description of any restrictions on data availability
- For clinical datasets or third party data, please ensure that the statement adheres to our policy

MIMIC-CXR data is available at https://physionet.org/content/mimic-cxr/2.0.0
PadChest data is available at https://bimcv.cipf.es/bimcv-projects/padchest
NIH ChestXray14 data is avaliable at https://nihcc.app.box.com/v/ChestXray-NIHCC/folder/36938765345

CheXpert data is available at https://aimi.stanford.edu/chexpert-chest-x-rays, and the official test data with labels is available at https://github.com/rajpurkarlab/cheXpert-test-set-labels.
ChestX-Det10 data is available at https://github.com/Deepwise-AILab/ChestX-Det10-Dataset.
Source data for figures are provided with this paper.

Human research participants

Policy information about studies involving human research participants and Sex and Gender in Research.

Reporting on sex and gender	This is not relevant to our study.
Population characteristics	This is not relevant to our study.
Recruitment	This is not relevant to our study.
Ethics oversight	This is not relevant to our study.

Note that full information on the approval of the study protocol must also be provided in the manuscript.

Field-specific reporting

Please select the one below that is the best fit for your research. If you are not sure, read the appropriate sections before making your selection.

[x] Life sciences [ ] Behavioural & social sciences [ ] Ecological, evolutionary & environmental sciences

For a reference copy of the document with all sections, see nature.com/documents/nr-reporting-summary-flat.pdf

Life sciences study design

All studies must disclose on these points even when the disclosure is negative.

Sample size	For the training dataset, we use the entire dataset, and for the evaluation datasets, we strictly follow the official train-test split to ensure fair comparisons and reproducible results. Our evaluation datasets cover almost all of the open-source data, and the sample size of the each dataset are: PadChest (n=39053 for 193 classes) , CheXpert (n=500 for 5 classes), NIH ChestXray14 (n=25596 for 14 classes), ChestX-Det10 (n=542 for 10 classes). By including these diverse datasets, we have ensured that our method's effectiveness is thoroughly validated across different scenarios and clinical settings.
Data exclusions	Data were not excluded from analysis.
Replication	Our experiment is carried out by programmers. We release all the codes to make sure all results are reproducible.
Randomization	In our experiment, we perform both zero-shot and fine-tuning settings. Specifically, in the zero-shot setting, we strictly follow the official train-test split, which are not random, so randomization is not relevant to your study.<br>In the fine-tuning setting, we randomly sample 90% for training, and 10% for validation from the official train set, and test on the official test set.
Blinding	Yes, the investigators were blinded to group allocation during data collection and analysis, as in our study, we use publicly available datasets and strictly follow the official train-test split, which are provided.

Reporting for specific materials, systems and methods

We require information from authors about some types of materials, experimental systems and methods used in many studies. Here, indicate whether each material, system or method listed is relevant to your study. If you are not sure if a list item applies to your research, read the appropriate section before selecting a response.

Materials & experimental systems

n/a	Involved in the study
☒	☐ Antibodies
☒	☐ Eukaryotic cell lines
☒	☐ Palaeontology and archaeology
☒	☐ Animals and other organisms
☒	☐ Clinical data
☒	☐ Dual use research of concern

Methods

n/a	Involved in the study
☒	☐ ChIP-seq
☒	☐ Flow cytometry
☒	☐ MRI-based neuroimaging
